# Correction: Potential model of *Scalesia pedunculata* carbon sequestration through restoration efforts in agricultural fields of Galapagos

**DOI:** 10.1371/journal.pone.0319749

**Published:** 2025-03-06

**Authors:** Nicolás Velasco, Patricia Jaramillo Diaz

The second author’s initials appear incorrectly in the citation. The correct citation is: Velasco N, Jaramillo P (2024) Potential model of Scalesia pedunculata carbon sequestration through restoration efforts in agricultural fields of Galapagos. PLoS ONE 19(5): e0302680. https://doi.org/10.1371/journal.pone.0302680.

In Figure 3, the Equation is missing on the figure. Please see the correct Figure 3 here.

**Fig 3:**
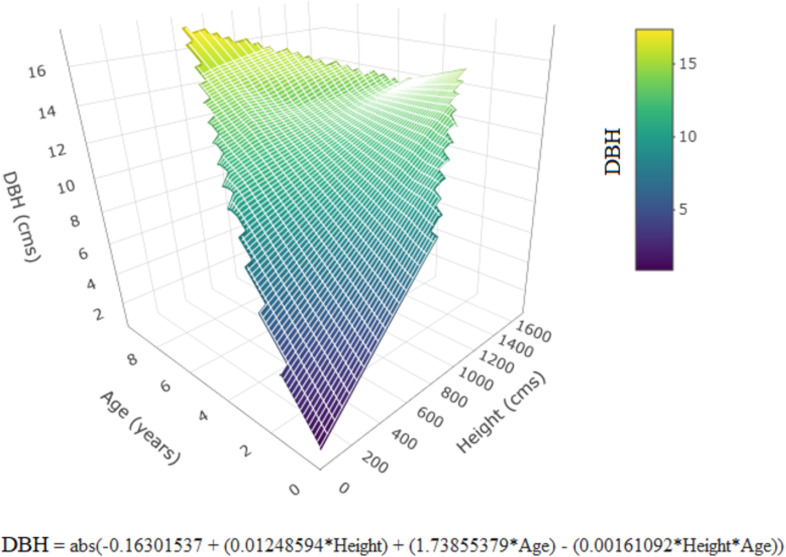
Estimated DBH as function of age and height. Equation included on the figure.
