# Supplementary figures and images for: Potential model of Scalesia pedunculata carbon sequestration through restoration efforts in agricultural fields of Galapagos
Source: PLoS One. 2024 May 16;19(5):e0302680. doi: 10.1371/journal.pone.0302680 (PMC11098518; doi:10.1371/journal.pone.0302680)

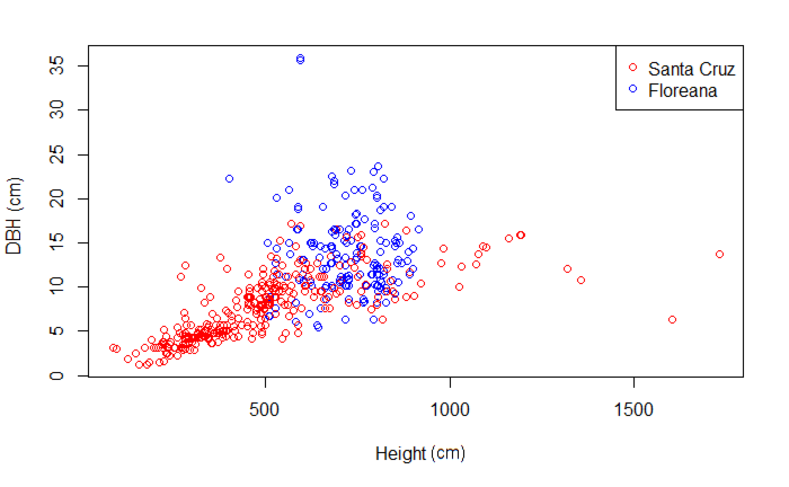

Supplement: S1 Fig — (TIF) [file pone.0302680.s001.tif]

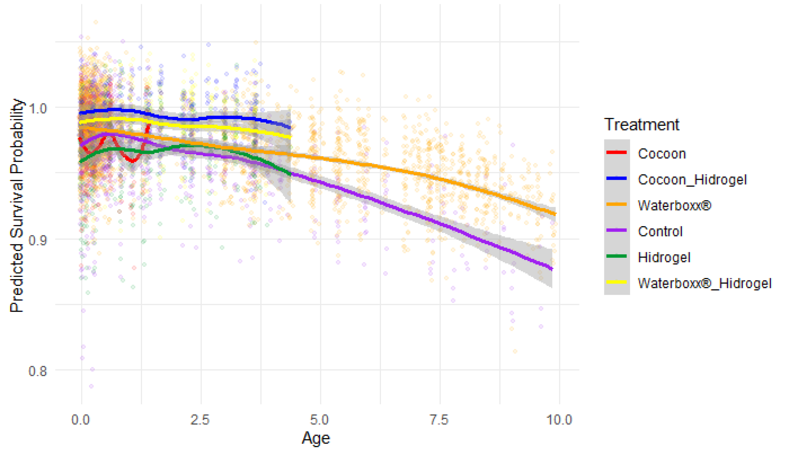

Supplement: S2 Fig — Grey intervals shows ±SE. (TIF) [file pone.0302680.s002.tif]
