# Supplementary material for: Potential model of Scalesia pedunculata carbon sequestration through restoration efforts in agricultural fields of Galapagos
Source: PLoS One. 2024 May 16;19(5):e0302680. doi: 10.1371/journal.pone.0302680 (PMC11098518; doi:10.1371/journal.pone.0302680)
Supplement: S2 Table — (DOCX) [file pone.0302680.s004.docx]

**Supplemental Figure 1** – Plot of the data gathered through the monitoring in each island


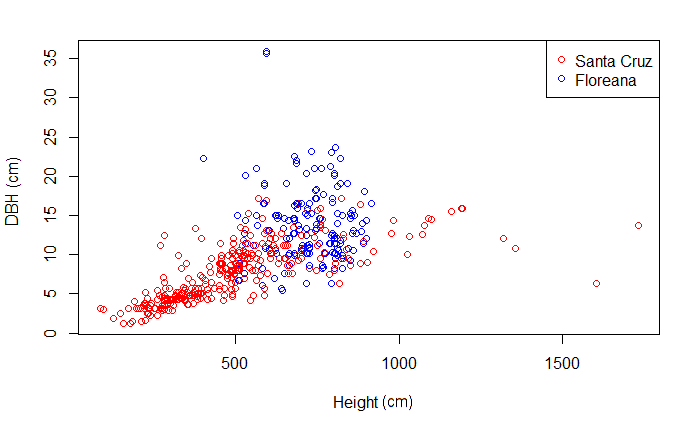


**Supplemental Figure 2** – Predicted survival by treatment. Grey intervals shows ±SE.


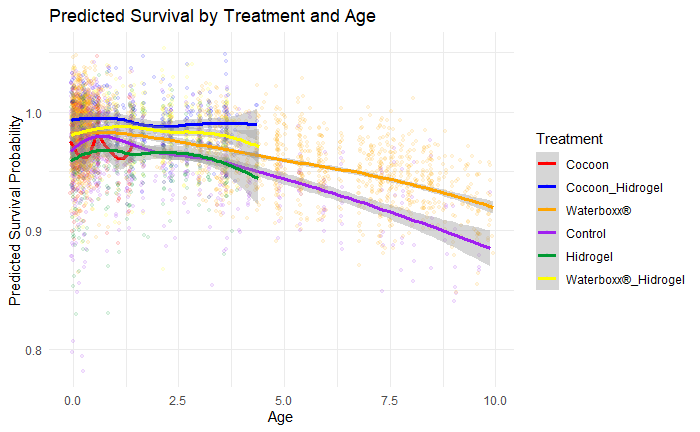


**Supplemental Table 1** - Names and information of the study sites. Sites 9 and 10 correspond to Floreana Island. Density = trees/ha

| **n** | **Site** | **Latitude** | **Longitude** | **Altitude** | **Ha** | **Trees** | **Density** |
| --- | --- | --- | --- | --- | --- | --- | --- |
| 1 | Los Gemelos | -0,63 | -90,39 | 605 | 0,014 | 7 | 500 |
| 2 | Darwin Ecogarden | -0,70 | -90,35 | 212 | 0,137 | 29 | 212 |
| 3 | Mario Piu' farm | -0,70 | -90,36 | 174 | 2,294 | 166 | 72 |
| 4 | El Chato Ranch | -0,67 | -90,43 | 242 | 0,109 | 11 | 101 |
| 5 | El Manzanillo Ranch | -0,70 | -90,40 | 170 | 0,205 | 10 | 49 |
| 6 | Terramar Ranch | -0,64 | -90,43 | 415 | 0,020 | 30 | 1500 |
| 7 | Mick farm | -0,70 | -90,32 | 190 | 0,025 | 7 | 280 |
| 8 | El Cascajo | -0,67 | -90,28 | 308 | 0,016 | 8 | 500 |
| 9 | Cerro Pajas | -1,30 | -90,46 | 392 | 0,003 | 4 | 1333 |
| 10 | Anibal San Miguel | -1,30 | -90,45 | 348 | 0,210 | 154 | 733 |

**Supplemental Table 2** - Inputs and equations to estimate the net contribution of transitioning coffee fields to agroforestry areas with *S. pedunculata.*

| **Inputs** |  |  |
| --- | --- | --- |
| Carbon Sequestration (10 y) | 21 | Mg/ha |
| Coffee hectares in Galapagos | 723 | ha |
| Galapagos Carbon Footprint | 532373 | CO_2_ Mg/y |
| Molecular weight C02 | 44.01 | g/mol |
| Molecular weight C | 12.01 | g/mol |
|  |  |  |
| **Eq 1 - Ratio C to C02** |  |  |
| 44,01/12,01=3664 |  |  |
|  |  |  |
| **Eq 2 - 10 y of *S. pedunculata* total sequestration** | | |
| 21 x 723 = 15183 Mg |  |  |
|  |  |  |
| **Eq 3 - Total Net Contribution (10 y)** | | |
| (15.183 x 3,664)/(523.373 x 10)*100 =**1.062%** | | |
