# Supplementary material for: Potential model of Scalesia pedunculata carbon sequestration through restoration efforts in agricultural fields of Galapagos
Source: PLoS One. 2024 May 16;19(5):e0302680. doi: 10.1371/journal.pone.0302680 (PMC11098518; doi:10.1371/journal.pone.0302680)
Supplement: Attachment — Submitted filename: PONE-D-23-41014 rev.pdf [file pone.0302680.s005.pdf]

PLOS ONE

Potential model of Scalesia pedunculata carbon sequestration through restoration efforts in agricultural fields of Galapagos.

--Manuscript Draft--

**Manuscript Number:**	PONE-D-23-41014
**Article Type:**	Research Article
**Full Title:**	Potential model of Scalesia pedunculata carbon sequestration through restoration efforts in agricultural fields of Galapagos.
**Short Title:**	Carbon sequestration model for Scalesia pedunculata
**Corresponding Author:**	Nicolás Velasco<br>Charles Darwin Foundation: Fundacion Charles Darwin<br>Puerto Ayora, ECUADOR
**Keywords:**	allometric equations; carbon footprint; carbon content; Brownian noise; tropical; highlands; coffee
**Abstract:**	Scalesia penduncculata Hook.f. is the dominant tree in several highlands' areas of the Ga-lapagos Archipelago, yet in inhabited islands the conversion to agricultural fields has reduced its cover. The transition to agroforestry systems including the species shows promising scenarios to restore its cover and to provide ecosystem services such as carbon sequestration. Here, based on fieldgathered data, we model the potential contribution of S. pedunculata stands in the carbon sequestration of Galapagos. Between 2013-2021, 426 S. pedunculata seedlings were planted in the highlands of Santa Cruz and Floreana islands using several restoration technologies, and their height and survival were monitored every three months. A subsample of 276 trees was used to estimate the DBH based on plant age and height. Based on scientific literature, biomass and carbon content were estimated across time. The final modeling included the density of plants in the restoration sites, estimated DBH, potential survival by restoration treatment, and a Brownian noise to add stochastic events. Overall, survival of S. pedunculata was high in control and slightly increased by most restoration treatments. A stand of 530 ind/ha was projected to sequester ~21 Mg C/ha in 10 years. If this is replicated over all Galapagos coffee production would contribute to the reduction of -1.062% of the Galapagos carbon footprint for the same period. This study adds to compiling benefits of restoring Galapagos flora.
**Order of Authors:**	Nicolás Velasco
	Patricia Jaramillo Diaz
**Additional Information:**	
**Question**	**Response**
**Financial Disclosure**<br><br>Enter a financial disclosure statement that describes the sources of funding for the work included in this submission. Review the submission guidelines for detailed requirements. View published research articles from *PLOS ONE* for specific examples.<br><br>This statement is required for submission and **will appear in the published article** if the submission is accepted. Please make sure it is accurate.	Yes

**Funded studies**<br>Enter a statement with the following details:<br>• Initials of the authors who received each award<br>• Grant numbers awarded to each author<br>• The full name of each funder<br>• URL of each funder website<br>• Did the sponsors or funders play any role in the study design, data collection and analysis, decision to publish, or preparation of the manuscript?<br><br>Did you receive funding for this work?	
Please add funding details.<br>as follow-up to "**Financial Disclosure**<br><br>Enter a financial disclosure statement that describes the sources of funding for the work included in this submission. Review the submission guidelines for detailed requirements. View published research articles from *PLOS ONE* for specific examples.<br><br>This statement is required for submission and **will appear in the published article** if the submission is accepted. Please make sure it is accurate.<br><br>**Funded studies**<br>Enter a statement with the following details:<br>• Initials of the authors who received each award<br>• Grant numbers awarded to each author<br>• The full name of each funder<br>• URL of each funder website<br>• Did the sponsors or funders play any role in the study design, data collection and analysis, decision to publish, or preparation of the manuscript?<br><br>Did you receive funding for this work?"	This research was funded by the “COmOn Foundation (Code: 1-63D-663)
Please select the country of your main research funder (please select carefully as in some cases this is used in fee	NETHERLANDS - NL

calculation).<br>as follow-up to "**Financial Disclosure**<br><br>Enter a financial disclosure statement that describes the sources of funding for the work included in this submission. Review the submission guidelines for detailed requirements. View published research articles from *PLOS ONE* for specific examples.<br><br>This statement is required for submission and **will appear in the published article** if the submission is accepted. Please make sure it is accurate.<br><br>**Funded studies**<br>Enter a statement with the following details:<br>• Initials of the authors who received each award<br>• Grant numbers awarded to each author<br>• The full name of each funder<br>• URL of each funder website<br>• Did the sponsors or funders play any role in the study design, data collection and analysis, decision to publish, or preparation of the manuscript?<br><br>Did you receive funding for this work?"	
**Competing Interests**<br><br>Use the instructions below to enter a competing interest statement for this submission. On behalf of all authors, disclose any competing interests that could be perceived to bias this work—acknowledging all financial support and any other relevant financial or non-financial competing interests.<br><br>This statement is required for submission and **will appear in the published article** if the submission is accepted. Please make sure it is accurate and that any funding sources listed in your Funding Information later in the submission form are also declared in your Financial Disclosure	The authors have declared that no competing interests exist.

statement.<br><br>View published research articles from *PLOS ONE* for specific examples.<br><br>**NO authors have competing interests**<br><br>Enter: *The authors have declared that no competing interests exist.*<br><br>**Authors with competing interests**<br><br>Enter competing interest details beginning with this statement:<br><br>*I have read the journal's policy and the authors of this manuscript have the following competing interests: [insert competing interests here]*<br><br>* typeset	
**Ethics Statement**<br><br>Enter an ethics statement for this submission. This statement is required if the study involved:<br><br>• Human participants<br>• Human specimens or tissue<br>• Vertebrate animals or cephalopods<br>• Vertebrate embryos or tissues<br>• Field research<br><br>Write "N/A" if the submission does not require an ethics statement.<br><br>General guidance is provided below. Consult the submission guidelines for detailed instructions. **Make sure that all information entered here is included in the Methods section of the manuscript.**	Before starting the restoration, we obtained verbal consent from farmers to use their fields. These areas were stipulated in the research permit PC-46-23, granted by the Galapagos National Park, the governing institution in the archipelago.

Powered by Editorial Manager® and ProduXion Manager® from Aries Systems Corporation

**Format for specific study types**<br><br>**Human Subject Research (involving human participants and/or tissue)**<br>• Give the name of the institutional review board or ethics committee that approved the study<br>• Include the approval number and/or a statement indicating approval of this research<br>• Indicate the form of consent obtained (written/oral) or the reason that consent was not obtained (e.g. the data were analyzed anonymously)<br><br>**Animal Research (involving vertebrate animals, embryos or tissues)**<br>• Provide the name of the Institutional Animal Care and Use Committee (IACUC) or other relevant ethics board that reviewed the study protocol, and indicate whether they approved this research or granted a formal waiver of ethical approval<br>• Include an approval number if one was obtained<br>• If the study involved *non-human primates*, add *additional details* about animal welfare and steps taken to ameliorate suffering<br>• If anesthesia, euthanasia, or any kind of animal sacrifice is part of the study, include briefly which substances and/or methods were applied<br><br>**Field Research**<br><br>Include the following details if this study involves the collection of plant, animal, or other materials from a natural setting:<br>• Field permit number<br>• Name of the institution or relevant body that granted permission	
**Data Availability**<br><br>Authors are required to make all data underlying the findings described fully available, without restriction, and from the time of publication. PLOS allows rare exceptions to address legal and ethical concerns. See the PLOS Data Policy and FAQ for detailed information.	No - some restrictions will apply

A Data Availability Statement describing where the data can be found is required at submission. Your answers to this question constitute the Data Availability Statement and **will be published in the article**, if accepted.<br><br>**Important:** Stating 'data available on request from the author' is not sufficient. If your data are only available upon request, select 'No' for the first question and explain your exceptional situation in the text box.<br><br>Do the authors confirm that all data underlying the findings described in their manuscript are fully available without restriction?	
**Describe where the data may be found in full sentences. If you are copying our sample text, replace any instances of XXX with the appropriate details.**<br><br>• If the data are **held or will be held in a public repository**, include URLs, accession numbers or DOIs. If this information will only be available after acceptance, indicate this by ticking the box below. For example: *All XXX files are available from the XXX database (accession number(s) XXX, XXX.*).<br>• If the data are all contained **within the manuscript and/or Supporting Information files**, enter the following: *All relevant data are within the manuscript and its Supporting Information files.*<br>• If neither of these applies but you are able to provide **details of access elsewhere**, with or without limitations, please do so. For example:<br><br>*Data cannot be shared publicly because of [XXX]. Data are available from the XXX Institutional Data Access / Ethics Committee (contact via XXX) for researchers who meet the criteria for access to confidential data.*<br><br>*The data underlying the results presented in the study are available from (include the name of the third party*	The data cannot be shared publicly as it is sourced from an ongoing restoration program and will be subject to further analysis by the owning institution in the future. Nonetheless, the data can be made available upon personal request to the contributing author

and contact information or URL).<br>• This text is appropriate if the data are owned by a third party and authors do not have permission to share the data.<br><br>* typeset	
Additional data availability information:	

Manuscript

Click here to access/download;Manuscript;Carbon_Paper_Upd.docx

Potential model of *Scalesia pedunculata* carbon sequestration through restoration efforts in agricultural fields of Galapagos.

Nicolás Velasco[1,2] and Patricia Jaramillo Diaz[1,3,*]

1 Charles Darwin Research Station, Charles Darwin Foundation, Puerto Ayora 200105, Ecuador

2 Departamento de Ciencias Ecológicas, Instituto de Ecología y Biodiversidad, Facultad de Ciencias, Universidad de Chile, Chile

3 Facultad de Ciencias, Universidad de Málaga, 29010 Málaga, Spain

* Correspondence: patricia.jaramillo@fcdarwin.org.ec

Abstract

Scalesia pendunculata Hook.f. is the dominant tree in several highlands' areas of the Galapagos Archipelago, yet in inhabited islands the conversion to agricultural fields has reduced its cover. The transition to agroforestry systems including the species shows promising scenarios to restore its cover and to provide ecosystem services such as carbon sequestration. Here, based on fieldgathered data, we model the potential contribution of *S. pedunculata* stands in the carbon sequestration of Galapagos. Between 2013-2021, 426 *S. pedunculata* seedlings were planted in the highlands of Santa Cruz and Floreana islands using several restoration technologies, and their height and survival were monitored every three months. A subsample of 276 trees was used to estimate the DBH based on plant age and height. Based on scientific literature, biomass and carbon content were estimated across time. The final modeling included the density of plants in the restoration sites, estimated DBH, potential survival by restoration treatment, and a Brownian noise to add stochastic events. Overall, survival of *S. pedunculata* was high in control and slightly

increased by most restoration treatments. A stand of 530 ind/ha was projected to sequester ~21 Mg C/ha in 10 years. If this is replicated over all Galapagos coffee production would contribute to the reduction of -1.062% of the Galapagos carbon footprint for the same period. This study adds to compiling benefits of restoring Galapagos flora.

Keywords: allometric equations, carbon footprint, carbon content, Brownian noise, tropical, highlands, coffee.

1. Introduction

Carbon sequestration has the potential to mitigate the impact of anthropogenic CO2 emissions on climate change, by transferring atmospheric CO2 to the plant tissues where it is stored [1]. Most long-term carbon sequestration is produced when plants decay, and recalcitrant compounds are stored in soil after microbial degradation [2]. However, in areas where plants are scarce or have been lost for an extended time, the recovery of the original woody and tall vegetation can substantially contribute to the initiation of the carbon sequestration process [3, 4]. Mature forests, for instance, can absorb and store large amounts of CO2 through photosynthesis [5].

In the Galapagos Archipelago, the development has led to the need for more land to sustain the population growth. Despite the Galapagos National Park established in 1979 a 3% of Galapagos surface as urban limits, major towns have increased in surface at an annual rate of 3.3% from 1992 to 2017 [6, 7], inside those limits. Historically, the original vegetation formations in the highland areas have been prone to more pressure to transform them into arable soils to sustain this growth [8]. Land in the agricultural zone of Santa Cruz for example, has increased over 67% since 1961, with a rampant increase in the

1960s and 1970s [9]. This replacement meant the reduction of the native forests and grasslands from 94% cover, to only 7% in 2018 in the non-protected areas [9].

Due to the population growth and the demographic expansion, the Galapagos Archipelago is not exempt of the worldwide issue of CO2 rise. The local government has taken some step ahead of the problem by supporting a neutral emissions airport [10], and adopting renewable energy systems aimed at achieving zero fossil fuel consumption for the electric generation [11]. Additionally, local studies have evaluated the contribution of native vegetation, ocean ecosystems and soil stocks as carbon sinks [12-14].

Scalesia pedunculata Hook.f., a member of the daisy family (Asteraceae), is a small tree that can grow up to 15 meters in height and is endemic to the Galapagos Archipelago [15, 16]. Despite being a pioneer species, the lack of late-successional species due to island isolation makes species of this genus the most frequent and dominant species in the higher elevations of the Galapagos islands [17, 18]. However, due to land transformation to agricultural fields and the spread of invasive species in the inhabited islands, the species' occupancy has been greatly reduced [19]. The recovery of its original cover has the potential of be an ally in the reduction of atmospheric CO2. For example, it has been estimated that mature stands of the species, reach at least 2500 individuals per hectare and have an aboveground biomass of 60 tons per hectare [18].

Since 2013, the ecological restoration program "Galapagos Verde 2050" (GV2050), of the Charles Darwin Foundation, has been working in the Galapagos Archipelago to restore the lost vegetation and conserve threatened plant species populations [20]. One of the projects involves the adoption of agroforestry systems in rural settlements by using native species combined with crops. This adds benefits through the ecosystem services provided by native flora, such as reduced abiotic stress and increase pollinators, and others. Specifically, the GV2050 has also been restoring *S. pedunculata* formations in

such agroforestry systems to evaluate the ecosystem services provided by the species, by also using different restoration tools aimed at boosting the survival and growth rate of the species. Many of these techniques have already shown positive results in other GV2050 restoration and agricultural initiatives [21-25]. The incorporation of *S. pedunculata* into coffee plantations holds promise for restoring landscapes where this species once thrived. Coffee typically benefits from the shade provided by taller plants to yield a higher quality product [26]. However, coffee plantations are often managed as monocultures. Consequently, integrating native plants to provide an additional height stratum can yield significant advantages. Furthermore, by restoring the dominant tree species in the highlands, we anticipate the recovery of their carbon sequestration function.

This study has two primary objectives: firstly, to assess how various restoration techniques impact the survival of *S. pedunculata*, and secondly, to evaluate the role of *S. pedunculata* in carbon sequestration when reintroduced into coffee plantations in the highlands of Santa Cruz and Floreana Islands. The study's findings aim to provide recommendations to Galapagos farmers regarding the potential benefits of transitioning from coffee monoculture to agroforestry systems incorporating native species. Additionally, it serves as an example of how such practices can contribute to the reduction of CO2 emissions, both locally and globally.

2. Materials and Methods

To develop a realistic carbon sequestration model for *S. pedunculata*, we undertook several steps, as depicted in Figure 1. This modelling framework is originally based on the restoration efforts, incorporating field-gathered data, supplemented by information and constraints from the literature. *2.1 Study area*

Since May 2013, farms in the highland areas of Santa Cruz and Floreana islands have been used as study sites to test their restoration with *S. pedunculata* (Fig. 2). Planting began mainly in 2013, and as new sites are continually added to the restoration initiative, the data for this study includes plantings up to September 2021. The highlands of both islands are characterized by a humid-tropical climate [27], with annual precipitation around 400-500 mm, and temperatures ranging annually between 24.2-33°C [28]. Soils are mostly loam [29]. Most dominant native species include *Scalesia pedunculata*, *Psidium galapagaeum* and *Zanthoxyllum fagara*, while agriculture and forestry have added exotic species such as *Cedrela odorata*, *Psidium guajava* and *Rubus niveus*, which have now become frequent elements in the highlands [30,31,32]. The restored areas with *S. pedunculata* in the ten study sites selected varied in area and density (Fig. 2; Appendix 1), and have as main activities the production of *Coffea arabica* and/or ecotourism. Before starting the restoration, we obtained verbal consent from farmers to use their fields. These areas were stipulated in the research permit PC-46-23, granted by the Galapagos National Park, the governing institution in the archipelago.

2.2 Planting

In each site, *S. pedunculata* seedlings of around 3 months (35.52±14.63 cm), were planted with one of the following methods: Groasis Waterboxx®, which is a plastic container that can store up-to 20L of water, to provide it to the plant through a wire and can be refilled with rainwater; Cocoon®, which is similar to Waterboxx® but does not refill and is made from cardboard, so degrades after a couple of years; and hydrogel, which is a polymer which can store up to 500 times its volume as water [33]. Both boxes were filled with 15L at the moment of planting, while 5L of water were added directly to the seedling in the planting hole. For hydrogel, 5 L of water were added to the planting hole, while 15L

of hydrated hydrogel were mixed with the soil. All treatments were compared to controls with 20L of water added directly to the planting hole. Additionally, in some study sites a combination of Cocoon plus hydrogel, or Waterboxx plus hydrogel were used, providing instead of 5L of water in the sowing hole, 5L of hydrated hydrogel. In total 426 *S. pedunculata* were planted between all islands, at a varying distance depending on the site conditions, reaching a mean density of 528 plants/ha between all sites (Appendix 1).

2.3 Growth and Survival Monitoring

Every three months the survival and height of each *S. pedunculata* plant was monitored in order to assess the restoration progress (Fig. 1). Data for this study included 5253 records scored until April 2023. Height was scored with measuring tape when they were young, while taller plants were scored with a marked bamboo cane to obtain exact measures. To obtain a function that can estimate the Diameter at Breast Height (DBH) of *S. pedunculata*, we selected a random subsample of 274 trees and measured their trunk circumference at breast height (Sup. Fig. 1). For trees with more than one trunk at the breast height, the radius of each individual branch was use to obtain their area, and the sum of areas was divided by π and the square root was taken to obtain a general radius that can represent the whole tree. Then, DBH was modelled as a linear function depending on height and age of the trees, and this function was used to estimate the DBH for each single monitoring (i.e., 5253 records). The linear correlation of our estimated DBH was assessed by obtaining the Pearson correlation coefficient against the age and height.

Survival of *S. pedunculata* trees was modelled through a generalized logistic mixed model, as a function of treatment and age, and using plant_ID and site as random effects, to account for the repeated measurements and site dependence (Sup. Fig. 1). Fixed effects were included only as additive terms to avoid overcomplexity, and because not all treatments were included in the same period of the restoration process, which hinders a

proper evaluation of individual effects interacting with age. The inclusion of the fixed terms was evaluated through a Wald Chi-square test, before testing the particular significance of levels within treatments. Survival trends were plotted up to 10 years using the predicted survival probabilities. Additionally, to evaluate treatment effects we used two time cut-offs available in our restoration data: 1 year, representing the adaptation period of seedlings after the first season after plantation; and 4 years, which represents when plant start to mature [14] and also is the cut-off for which most treatments have sufficient data. Analyses were performed in RStudio, with R 4.2.2 [34] and packages lme4 [35] and car [36].

2.4 DM Allometry & Carbon Sequestration Model

To obtain the dry mass of *S. pedunculata*, we used the functions from [18] (Equations 1 & 2; Fig. 1), which decompose the biomass of *S. pedunculata* trees into two components: trunk plus branches (i.e., hard woody structures) and foliage (i.e., photosynthetic tissues).

$$\text{Eq1}\rightarrow \text{DryMass (woody)}=(0.01540*(\text{DBH}^2)^{1.60906})$$

$$\text{Eq2}\rightarrow \text{DryMass (foliage)}= (0.01769*(\text{DBH}^2)^{0.77946})$$

Most studies that evaluate plants' carbon content in dry biomass, exclude a varying percentage between 1.3-2.5% of volatile C [37], which should be considered as carbon sequestration in living tissues. Thus, the selected functions, which include the scoring of leaves (i.e., alive tissues), partially overcome this limitation. Additionally, 50% is used as the standard value for carbon content on plant dry biomass. Yet, several studies agree there is great variation depending on type of tree species (i.e., evergreen vs deciduous, or conifer vs broad-leaved) and climate-latitude where they grow [37-39]. Here, we used a 48%, as the literature indicates that carbon content in tropical broad-leaves species ranges from 47-49% .

Our carbon sequestration model (CSM) was analyzed using all available data, as well as subsets of the two treatments with more extensive data: Controls and Waterboxx. We used 10 years as cut-off to explore the CSM, as this period is a realistic time considering a restoration effort. CSM was constructed using the [18] equations, using the 48% of carbon content in biomass, and using a density of 530 trees/ha (Fig. 1) based on the mean density that we used on our study sites (Appendix 1). The literature reports that natural stands of *S. pedunculata* varied in densities, ranging between 2.5 to 11.6 thousand plants per hectare [18,40]. This implies that the density we used is equivalent to a realistic recommendation for farmers, suggesting the reincorporation of approximately 4.5 to 21% of the natural density. The temporal projection of the CSM was achieved by using the survival probabilities estimated earlier, accounting that not all trees might survive in a restoration effort. Additionally, to consider stochastic events that can reduce or increase the population size (e.g., bad weather conditions in some years, or on the opposite, unmanaged *S. pedunculata* regeneration at the farm scale), we included in the projection a Brownian noise that can account for a random walk in the trend of carbon sequestration (Fig. 1). For this, a 20% of random deviation was included as Brownian motion, in line with similar levels of uncertainty used in related approaches [41].

3. Results

The estimated DBH showed a high Pearson correlation coefficient with age (0.919) and height (0.967), indicating that DBH can be effectively expressed as a linear function of these two variables (Fig. 3). The derived equation showed that both age and height positively influenced DBH; taller plant of the same age, as well as older plant of the same height, consistently exhibited higher DBH.

The survival model showed that treatment and age were significant in predicting the survival outcome (both p<0.001; Table 1). Survival of plants during the first four years improved specially with both treatments that included a combination of two technologies (Fig. 4), and for all treatments the survival dropped only slightly after 4 years. When considering the all data model, albeit some differences in the trends of Waterboxx and Controls (Sup. Fig. 2), only Cocoon plus Hydrogel showed to be different from the rest of the treatments (Table 1; p=0.043). However, data for several treatments remain insufficient to explore if this trend persist over longer restoration periods (Sup. Fig. 2).

According to the CSM a single tree of *S. pedunculata* can store up to 37-39 kg of carbon, over a time span of 10 years. Additionally, there is a trend change in the carbon sequestration mediated by the height and survival of treatments across time, where the restoration technique with the most extensive data (Waterboxx) starts to provide very slight increases in carbon sequestration after 5 years (Fig. 5). Overall, when including stochastic events, both the controls and Waterboxx treatments were estimated to reach both around ~21 Megagrams of assimilated carbon in stands of 530 trees/ha.

4. Discussion

Scalesia pedunculata has experienced significant habitat loss in the highlands of the Galapagos [19]. Efforts to restore this species through the GV2050 Program commenced in 2013, initially focusing on identifying the most effective restoration methodologies [20]. Our findings suggest an incremental improvement in plant survival over the initial four years, particularly when utilizing technologies such as Waterboxx + Hydrogel (see Supplementary Figure 2) and, notably, Cocoon + Hydrogel (refer to Table 1). This implies that biodegradable technologies, adaptable to the unique highland conditions of the

Galapagos, may offer significant advantages. Such technologies have already demonstrated positive outcomes in enhancing the survival and growth of other species in similar ecosystems [42-44]. Despite these promising results, it's important to note that our comparative analysis is currently limited to a four-year timeframe due to the staggered introduction of different treatments in the restoration program. Consequently, there is a critical need for ongoing and extended research to verify whether these positive trends persist in the long term, thereby ensuring the effective and sustainable restoration of *Scalesia pedunculata* in the Galapagos highlands.

For longer timespans we can only make comparisons between controls and Waterboxx, which despite increasing slightly the survival of *S. pedunculata* (Sup. Fig. 2), do not translate into substantial gains in the carbon sequestration (Fig. 5). It should be noted that the lack of significant differences in the long run by Waterboxx, or between several treatments at the 4-year cut-off, applies only to survival. Here we do not focus on the architecture of the *S. pedunculata* plants and although that Waterboxx tends to strengthen roots on plants [45], we do not know how these treatments specifically affect the *S. pedunculata* roots system. A robust root system is undoubtedly vital for *S. pedunculata* in its natural habitat. This is particularly evident when observing numerous mature plants succumbing to various factors, such as root rot or waterlogging, especially during severe El Niño events [46]. Then, we cannot rule out that the restoration techniques could have later positive impacts on the root system or on these mass mortality events.

Similarly, it is important to note that the input provided to the carbon model included differentiated heights and survival by each treatment to model aboveground sequestration, but the literature mentions that around half of the sequestered carbon is on the root system in most forest types [47]. Some specific attempts have been made to model below-ground carbon sequestration in *S. pedunculata*. For example, belowground

biomass has been estimated at around 15% given the shallow root system, although carbon sequestration models that account for belowground biomass are still too incipient (R2=0.22) [48] and insufficient to produce reliable models. From the above, we can recommend that additional new research should investigate the contribution of the root system of *S. pedunculata*.

This study provides a modelling framework to advise coffee farmers on one of the potential benefits of including *S. pedunculata* into their fields. Currently, the area cultivated with coffee in Galapagos is around 723 ha [49], with a potential to store up to 15 x 103 Megagrams of assimilated carbon. The CO2 footprint of Galapagos is mainly produced by tourism and has been estimated to be 523 x103 Mg/year [50,51]. This means the transition to agroforestry systems that include native species such as *S. pedunculata* could reduce the local footprint by -1.062% in a timeframe of 10 years (Appendix 2). There is a promising opportunity for such initiatives. It is predicted that in the Galapagos Archipelago, the increase in highlands rainfall due to climate change will lead to a significant increase in soil carbon content [12]. Thus, restoration effort that goes in the direction of increasing native cover could adhere to that trend.

Albeit this is a modelling approach, which in turn can have reduced realism, several constraints were included to make it more plausible. Here we used survival estimates and heights from field-based data, while density was based on low numbers compared to the ones in natural stand [40]. Moreover, 48% carbon content in biomass has been shown to be more grounded for tropical forest species [39], and the addition of a Brownian noise to account for stochastic uncertainty follows a similar approach to that in studies aimed to increase realism to soil carbon sequestration [41,52]. One limitation in our study is the time frame used (i.e., 10 years), as we only have data for that period. Literature has shown that *S. pedunculata* can live longer, up to 15-20 years [15,16]. Additionally, our estimated

DBH reached a maximum of 17.61 cm, while real data of the subsample method show several maximum values between 20-23 cm, which are in tune with the literature [16,17]. From the limitations exposed, plus the exclusion of the belowground biomass, we can infer that the models provided here are underestimating the carbon sequestration of *S. pedunculata*.

New research is needed to develop better carbon sequestration models in the Galapagos. The number of *S. pedunculata* trees being used by the GV2050 has almost doubled during 2022/2023 by including several new restoration sites in Floreana, and also by now including San Cristobal Island. The continuous monitoring of the trees and the gathering of additional data will support a better understanding in the future of the carbon sequestration dynamics in the Archipelago. Still, the study can already be used to suggest that farmers transition to more sustainable practices and serve as a model for replication in other tropical regions.

Funding: This research was funded by the “COmOn Foundation (Code: 1-63D-663).

Institutional Review Board Statement: This study was conducted and approved by the Institutional Review Board of Charles Darwin Research Station.

Data Availability Statement: Further information is available at: https://gv2050.shinyapps.io/GV2050-restoR/; http://www.galapagosverde2050.com/, accessed on 11 October 2023.

Acknowledgments: We are in debt to the farmers that allowed us to take data from their farms, the Floreana Junta Parroquial, the Santa Cruz ‘farmers’ cooperative, the DPNG (Dirección del Parque Nacional Galapagos), and the Agriculture Ministry of Galápagos (MAG). We highly appreciate the comments of our scientific advisers, Washington Tapia and James P. Gibbs. We thank David Cevallos, Anna Calle-Loor, Pavel Enríquez-Moncayo, Paúl Mayorga, Danyer Zambrano and David Cárdenas, as well to all national and international volunteers for their valuable contribution gathering data for this article. This publication is contribution number 2584 of the Charles Darwin Foundation for the Galapagos Islands.

Conflicts of Interest: The authors declare no conflicts of interest.

References

1. Lal, R., Negassa, W., & Lorenz, K. Carbon sequestration in soil. Curr Opin Environ Sustain. 2015, 15, 79-86. [CrossRef]

2. Whalen, J. K., Gul, S., Poirier, V., Yanni, S. F., Simpson, M. J., Clemente, J. S., .Feng, X., Grayston, S. J., Barker, J., Gregorich, E. G., Angers, D. A., Rochette, P. & Janzen, H. H. (2014). Transforming plant carbon into soil carbon: Process-level controls on carbon sequestration. Can. J. Plant Sci. 2014. 94(6), 1065-1073. [CrossRef]

3. De Deyn, G. B., Shiel, R. S., Ostle, N. J., McNamara, N. P., Oakley, S., Young, I., Freeman, C., Fenner, N., Quirk, H., & Bardgett, R. D. Additional carbon sequestration benefits of grassland diversity restoration. J. Appl. Ecol. 2011, 48(3), 600-608. [CrossRef]

4. Hu, L., Li, Q., Yan, J., Liu, C., & Zhong, J. Vegetation restoration facilitates belowground microbial network complexity and recalcitrant soil organic carbon storage in southwest China karst region. Sci. Total Environ. 2022, 820, 153137. [CrossRef]

5. Wheeler, C. E., Omeja, P. A., Chapman, C. A., Glipin, M., Tumwesigye, C., & Lewis, S. L. (2016). Carbon sequestration and biodiversity following 18 years of active tropical forest restoration. Forest ecology and management, 373, 44-55.

6. Sánchez, G. R. (2007). Population Dynamics in the towns of the Galapagos Islands: A G.I.S. Approach: A case study of Puerto Ayora town, Santa Cruz Island (Issue September). Vrije Universiteit Brussel.

7. Benítez, F. L., Mena, C. F., & Zurita-Arthos, L. (2018). Urban land cover change in ecologically fragile environments: the case of the Galapagos Islands. Land, 7(1), 21.

8. Laso, F. J., Benítez, F. L., Rivas-Torres, G., Sampedro, C., & Arce-Nazario, J. (2019). Land cover classification of complex agroecosystems in the non-protected highlands of the Galapagos Islands. Remote sensing, 12(1), 65.

9. Alomía Herrera, I., Paque, R., Maertens, M., & Vanacker, V. (2022). History of Land Cover Change on Santa Cruz Island, Galapagos. Land, 11(7), 1017. [CrossRef]

10. Cáceres, D. V. G. (2018). Un aeroport neutre en carbone essentiel a la protection environnementale de Galápagos. Programme ERC n° 340770 Human Sea. Pp 1-7

11. Eras-Almeida, A. A., Egido-Aguilera, M. A., Blechinger, P., Berendes, S., Caamaño, E., & García-Alcalde, E. (2020). Decarbonizing the Galapagos Islands: Techno-economic perspectives for the hybrid renewable minigrid Baltra–Santa Cruz. Sustainability, 12(6), 2282.

12. Rial, M., Cortizas, A. M., Taboada, T., & Rodríguez-Lado, L. (2017). Soil organic carbon stocks in Santa Cruz Island, Galapagos, under different climate change scenarios. Catena, 156, 74-81.

13. Costa, M. T., Salinas-de-León, P., & Aburto-Oropeza, O. (2019). Storage of blue carbon in isolated mangrove forests of the Galapagos' rocky coast. Wetlands ecology and management, 27(4), 455-463.

14. Tanner, M. K., Moity, N., Costa, M. T., Jarrin, J. R. M., Aburto-Oropeza, O., & Salinas-de-León, P. (2019). Mangroves in the Galapagos: ecosystem services and their valuation. Ecological economics, 160, 12-24.

15. Hamann, O. (2001). Demographic studies of three indigenous stand-forming plant taxa (*Scalesia*, *Opuntia*, and *Bursera*) in the Galápagos Islands, Ecuador. Biodiversity & Conservation, 10, 223-250.

16. Runkle, J. R., & Runkle, W. A. (2005). Structure and development of a *Scalesia pedunculata* stand in the Galapagos Islands. Galapagos research 63, 12-15.

17. Itow, S. (1995). Phytogeography and Ecology of *Scalesia* (Compositae) Endemic to the Galapagos Islands. Pacific Science, 49(1), 17–30.

18. Kitayama, K., & Itow, S. (1999). Aboveground biomass and soil nutrient pools of a *Scalesia pedunculata* montane forest on Santa Cruz, Galápagos. Ecological research, 14, 405-408.

19. Rentería, J. L., Gardener, M. R., Panetta, F. D., Atkinson, R., & Crawley, M. J. (2012). Possible impacts of the invasive plant Rubus niveus on the native vegetation of the *Scalesia* forest in the Galapagos Islands. PLoS One, 7(10), e48106

20. Jaramillo, P., Lorenz, S., Ortiz, G., Cueva, P., Jiménez, E., Ortiz, J., Rueda, D., Freire, M., Gibbs, J. P., & Tapia, W. (2013). Galápagos Verde 2050: an opportunity to restore degraded ecosystems and promote sustainable agriculture in the Archipelago. Biodiversity and ecosystem restoration: GNPD, GCREG, CDF, and GC. Galápagos Report, 2014, 133-143.

21. Tapia, P. I., Negoita, L., Gibbs, J. P., & Jaramillo, P. (2019). Effectiveness of water-saving technologies during early stages of restoration of endemic Opuntia cacti in the Galápagos Islands, Ecuador. PeerJ, 7, e8156.

22. Negoita, L., Gibbs, J. P., & Jaramillo, P. (2022). Cost- effectiveness of water-saving technologies for restoration of tropical dry forest: a case study from the Galapagos Islands, Ecuador. Restoration ecology, 30(5), e13576.

23. Jaramillo, P., Cornejo, F., Solís, M., Mayorga, P., & Negoita, L. (2023). Effect of water-saving technologies on productivity and profitability of tomato cultivation in Galapagos, Ecuador. J. Appl. Hortic, 24, 296-301.

24. Jaramillo, P., Calle-Loor, A., Gualoto, E., Bolaños, C., & Cevallos, D. (2022). Adoption of Sustainable Agriculture Practices through Participatory Research: A Case Study on Galapagos Islands Farmers Using Water-Saving Technologies. Plants, 11(21), 2848.

25. Jaramillo, P., Calle-Loor, A., Velasco, N., & Cevallos, D. (2023). Water-Saving Technologies in Galapagos Agriculture: A Step towards Sustainability. Horticulturae, 9(6), 683.

26. Albertin, A., & Nair, P. K. R. (2004). Farmers' perspectives on the role of shade trees in coffee production systems: An assessment from the Nicoya Peninsula, Costa Rica. Human Ecology, 32, 443-463.

27. Beck, H. E., Zimmermann, N. E., McVicar, T. R., Vergopolan, N., Berg, A., & Wood, E. F. (2018). Present and future Köppen-Geiger climate classification maps at 1-km resolution. Scientific data, 5(1), 1-12.

28. Fick, S. E., & Hijmans, R. J. (2017). WorldClim 2: new 1- km spatial resolution climate surfaces for global land areas. International Journal of Climatology, 37(12), 4302-4315.

29. Poggio, L., De Sousa, L. M., Batjes, N. H., Heuvelink, G., Kempen, B., Ribeiro, E., & Rossiter, D. (2021). SoilGrids 2.0: producing soil information for the globe with quantified spatial uncertainty. Soil, 7(1), 217-240.

30. Bimbosa, I., & del Consuelo, M. (2019). Caracterización de la composición botánica dentro de una plantación modelo de café bajo un sistema agroforestal en la zona de El Camote, Isla Santa Cruz, Galápagos (Bachelor's thesis, Quito: UCE).

31. CGREG. (2016). Plan Galápagos: Plan de desarrollo sustentable y ordenamiento territorial del régimen especial de Galápagos 2015-2020 (CGREG & MINTUR (eds.). Pp 275.

32. Lundh, B. J. P. (1965). The farm area and cultivated plants on Santa Cruz, 1932 – 1965, with remarks on other parts of Galapagos. Galapagos Research, 64, 12–25.

33. Cosecha Lluvia. Cosecha de Lluvia: El Primer Hidroretenedor en Ecuador Con Hidrogel. Cosecha de Lluvia. Innovación Futurista. 2015. Available online: https://www.cosechadelluvia.com/cosecha-de-lluvia-conoce-mas (accessed on 7 May 2023).

34. R Core Team (2022). R: A language and environment for statistical computing. R Foundation for Statistical Computing, Vienna, Austria. URL https://www.R-project.org/.

35. Bates, D., Mächler, M., Bolker, B., & Walker, S. (2015). "Fitting Linear Mixed-Effects Models Using lme4." Journal of Statistical Software, 67(1), 1–48. doi: 10.18637/jss.v067.i01.

36. Fox, J., & Weisberg, S. (2019). An R Companion to Applied Regression, Third edition. Sage, Thousand Oaks CA. https://socialsciences.mcmaster.ca/jfox/Books/Companion/.

37. Thomas, S. C., & Martin, A. R. (2012). Carbon content of tree tissues: a synthesis. Forests, 3(2), 332-352.

38. IPCC. Forest lands. Intergovernmental Panel on Climate Change Guidelines for National Greenhouse Gas Inventories; Institute for Global Environmental Strategies (IGES): Hayama, Japan, 2006; Volume 4, p. 83.

39. Ma, S., He, F., Tian, D., Zou, D., Yan, Z., Yang, Y., Zhou, T., Shen, H. & Fang, J. (2018). Variations and determinants of carbon content in plants: a global synthesis. Biogeosciences, 15(3), 693-702.

40. Hamann, O. (1979). Dynamics of a stand of *Scalesia pedunculata* Hooker fil., Santa Cruz Island, Galápagos. Botanical Journal of the Linnean Society, 78(2), 67-84.

41. Kim, M. K., & McCarl, B. A. (2009). Uncertainty discounting for land-based carbon sequestration. Journal of Agricultural and Applied Economics, 41(1), 1-11. [CrossRef]

42. Fajardo, L., Rodríguez, J. P., González, V., & Briceño-Linares, J. M. (2013). Restoration of a degraded tropical dry forest in Macanao, Venezuela. Journal of Arid Environments, 88, 236-243.

43. Mohsin, F. (2020). Species-specific responses to restoration interventions in a Tamaulipan thorn forest (Chapter II). In Optimizing Species Selection for Forest Restoration in the Lower Rio Grande Valley (Doctoral dissertation). The University of Texas Rio Grande Valley.

44. Carabassa, V., Alba- Patiño, D., García, S., Campo, J., Lovenstein, H., Van Leijen, G., Castro, A., Gonzáles, F., Viera, G. & Alcañiz, J. M. (2021). Water- saving

techniques for restoring desertified lands: Some lessons from the field. Land Degradation & Development, 33(1), 133-144. [CrossRef]

45. Groasis. (2023). Diez veces reutilizable Waterboxx® plant cocoon. https://www.groasis.com/es/tecnologia/el-plantcocoon-la-planta-capullo-del-groasis-waterboxx-plantcocoon-tiene-un-porcentaje-de-supervivencia-del-90-y-un-90-menos-de-consumo-de-agua. Accessed 21-11-23.

46. Itow, S., & Mueller-Dombois, D. (1988). Population structure, stand-level dieback and recovery of *Scalesia pedunculata* forest in the Galapagos Islands. Ecological Research, 3(3), 333-339.

47. Montagnini, F., & Nair, P. R. (2004). Carbon sequestration: an underexploited environmental benefit of agroforestry systems. In New Vistas in Agroforestry: A Compendium for 1st World Congress of Agroforestry, 2004 (pp. 281-295). Springer Netherlands

48. Stern, M., Olander, J., & Cárdenas, S. (2012). Evaluation of carbon stock of *Scalesia* in coffee agroforestry systems on Santa Cruz Island, Galapagos, Ecuador. Prepared for Conservación Internacional, Ecuador.

49. Ministerio de Agricultura y Ganadería. (2022). Informe de gestión: Período 1 de enero al 31 de diciembre de 2022. Dirección Distrital de Galápagos.

50. Cordova-Vallejo, X. M., Blanco, E. E., Yang, X., & Ponce-Cueto, E. (2012). Carbon Footprint of the Galapagos Islands–Quantifying the Environmental Impact of Tourist Activities.

51. Platts, E. J., Keifer, H. L., & Samuels, K. L. (2023). Carbon footprints of travel to World Heritage Sites: communicating climate to potential tourists through a consumption-based life-cycle assessment. Journal of Heritage Tourism, 1-20.

52. Jones, J. W., Koo, J., Naab, J. B., Bostick, W. M., Traore, S., & Graham, W. D. (2007). Integrating stochastic models and in situ sampling for monitoring soil carbon sequestration. Agricultural systems, 94(1), 52-62. DOI: https://doi.org/10.1016/j.agsy.2005.06.023

Figure 1 – Modelling approach for estimating carbon sequestration by *S. pedunculata* stands. First, gathering of field data acts as the initial input, used to estimate DBH as function of height and age, and to assess the potential of restoration technologies in increasing the survival *S. pedunculata* plants. Literature information is used to: obtain an allometric function to estimate the dry mass of *S. pedunculata*, and the percentage of carbon content in tropical broad-leaves species. Final model is derived using a low density of plants, based on the study sites, and incorporates a Brownian noise to account for population stochasticity.

Figure 2 – Location of the study sites (triangles). Blue triangles indicate the study sites that were subsampled to score the DBH of *S. pedunculata*. The agricultural zones of Santa Cruz and Floreana are delimited in orange. The green gradient denotes the increase in altitude. The greener, the higher altitude. For the names of study sites and their specific areas, see supplemental Table 1. Site 1 is a natural site but was included because it had the few older and taller *S. pedunculata* specimens planted (Appendix A).

Figure 3 – Estimated DBH as function of age and height. Equation included on the figure.

Figure 4 – Estimated survival of *S. pedunculata* for two times cut-off (1 and 4 years) by treatment.

Figure 5 - Megagrams of carbon sequestration for a stand of 530 *S. pedunculata* trees/ha. Models based on predicted survivals and heights, by treatments over time. In black is also depicted the trend for the full dataset. All trends include a Brownian noise of 20% deviation. Grey intervals show ±SE.

Table 1 – Results for the Wald Chi-square test and summary of the survival model. Std. Dev = standard deviation, Std. E = standard error. Significant values (p ≤0.05) in bold.

Wald Chisq Test

Term	Chisq	DF	*p*
Intercept	34,609	1	**<0,001**
Treatment	11,681	5	**0,0394**
Age	22,518	1	**<0,001**

Random effects

Groups	Variance	Std. Dev
Plant_ID	0,812	0,901
Site	0,994	0,997

Fixed effects

	Estimate	Std. E	z	*p*
Intercept	3,932	0,668	5,883	**<0,001**
Cocoon + Hydrogel	1,973	0,975	2,023	**0,043**
Control	-0,223	0,589	-0,379	0,705
Hydrogel	-0,333	0,609	-0,547	0,584
Waterboxx®	0,264	0,626	0,422	0,673
Waterboxx® + Hydrogel	0,711	0,701	1,014	0,311
age	-0,234	0,049	-4,745	**<0,001**

Click here to access/download;Figure;Fig 5.png

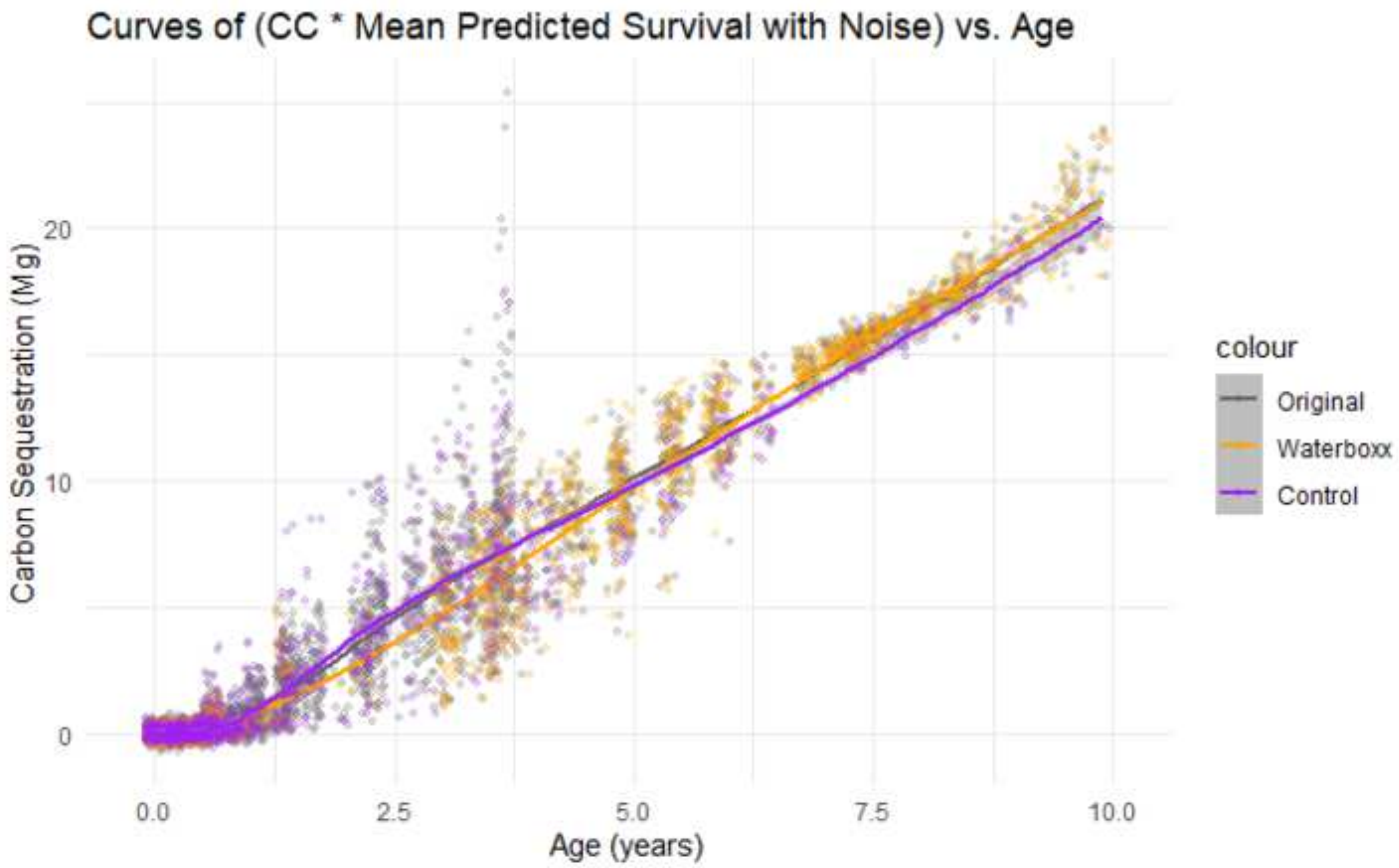

 Click here to access/download;Figure;amended figurine.png

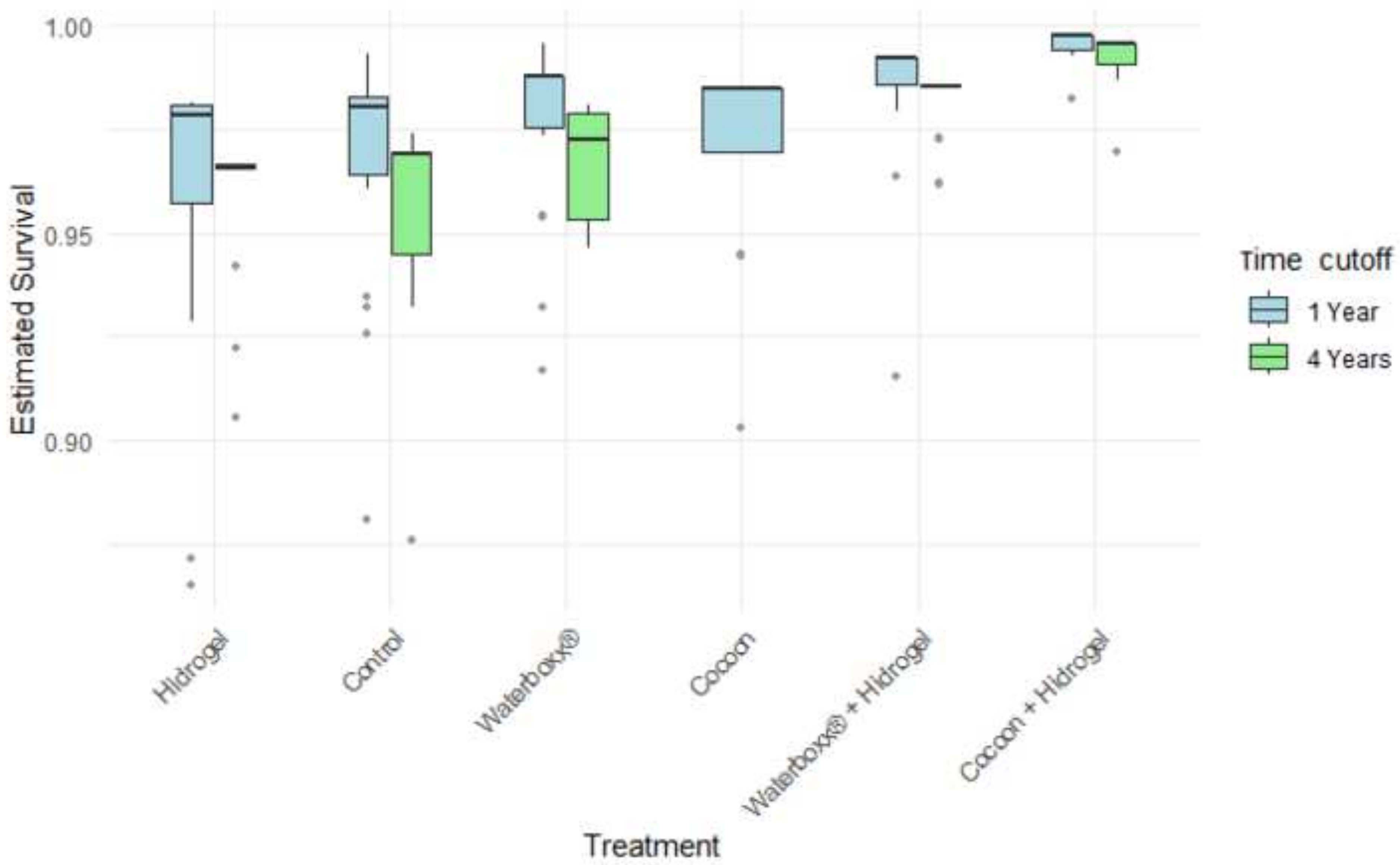

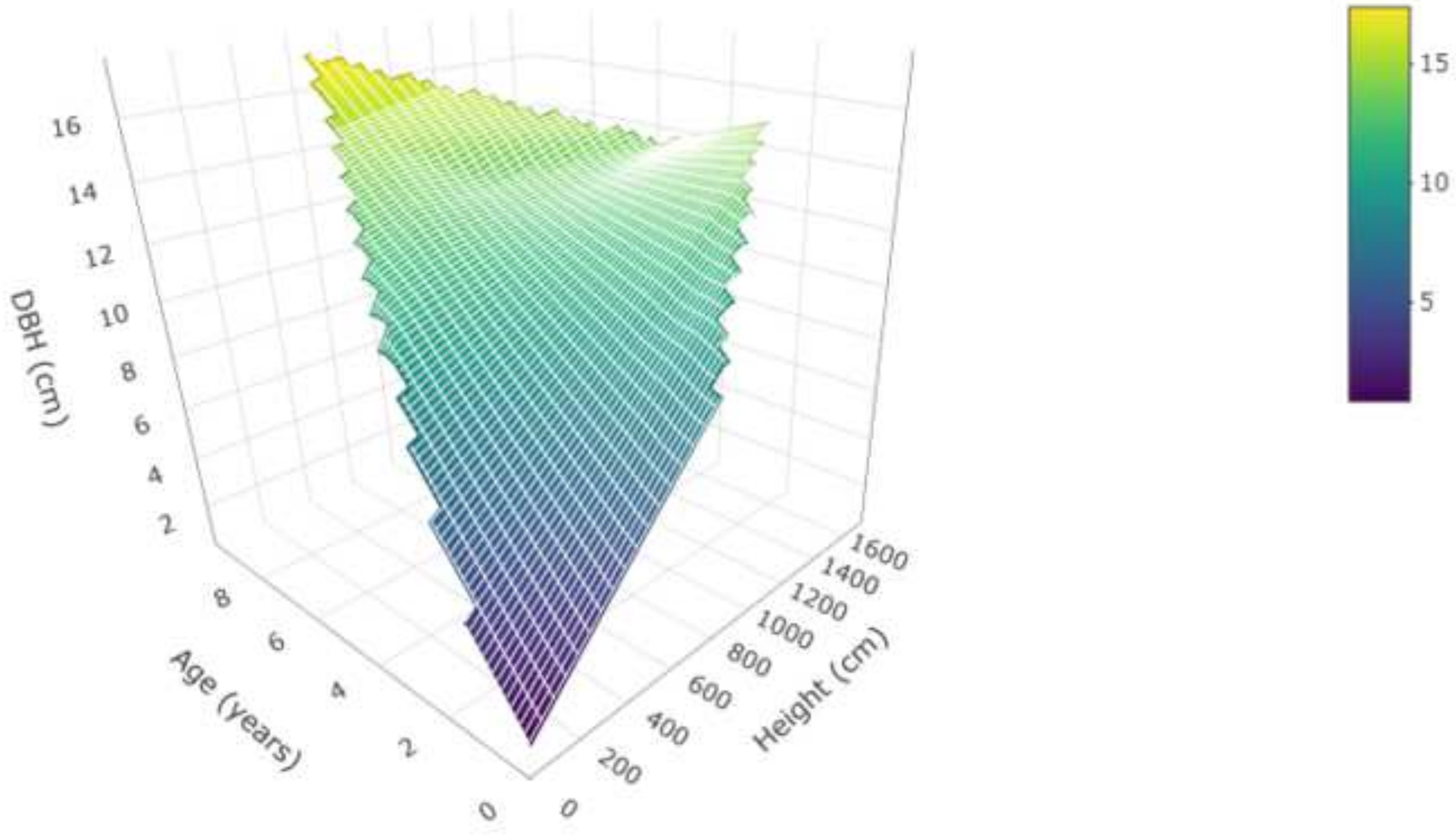
DBH (cm)
16
14
12
10
8
6
4
2
Age (years)
8
6
4
2
0
Height (cm)
0
200
400
600
800
1000
1200
1400
1600
15
10
5

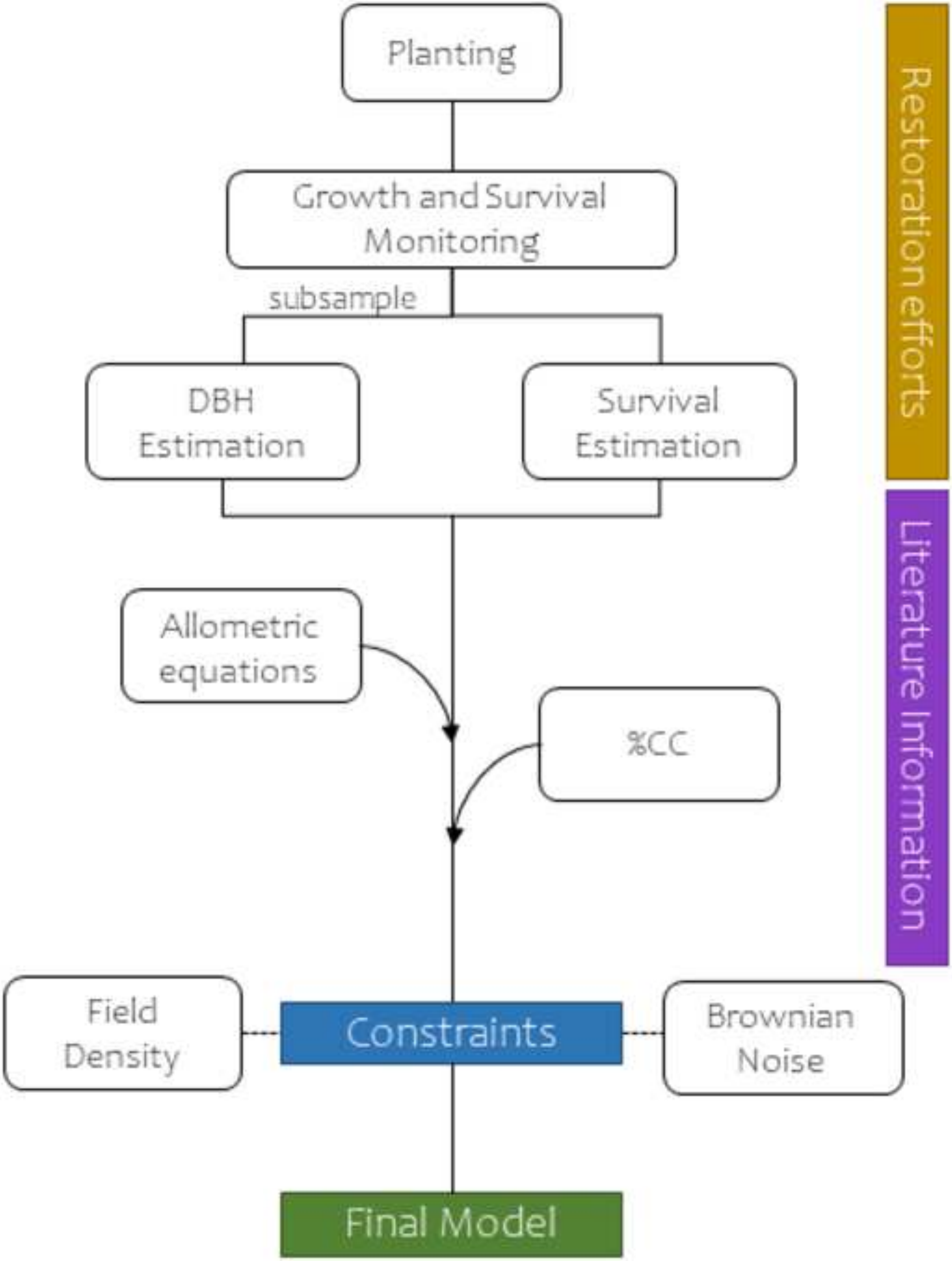
Planting
Growth and Survival Monitoring
subsample
DBH Estimation
Survival Estimation
Allometric equations
%CC
Field Density
Constraints
Brownian Noise
Final Model
Restoration efforts
Literature Information

Figure 2 - HQ

Click here to access/download;Figure;Mapa.tif

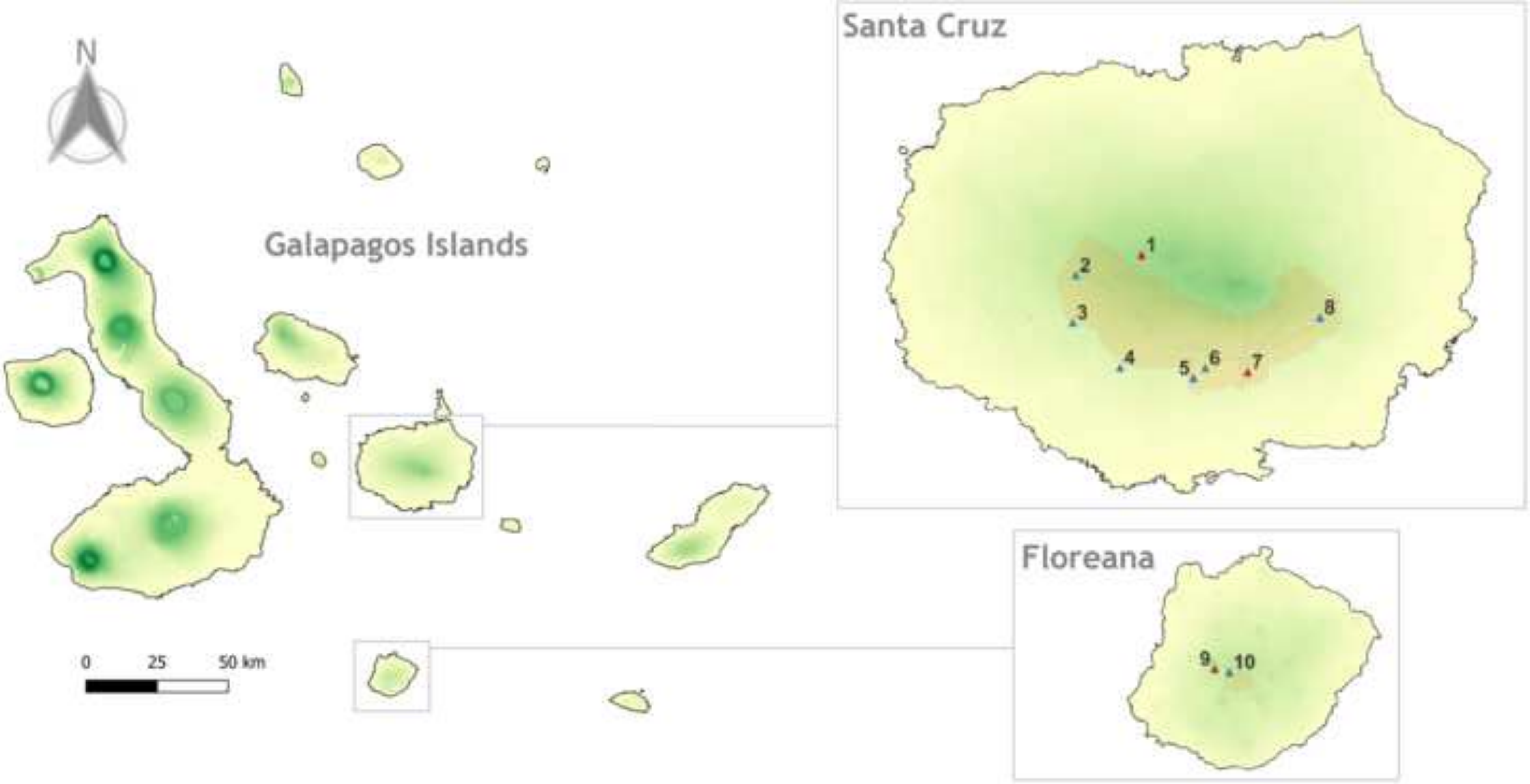

Supplemental

Click here to access/download
Supporting Information
Supplemental Material_Upd.docx

Click here to access/download
Supporting Information - Compressed/ZIP File Archive
Permit PC-46-23-signed.pdf
