# Supplementary material for: Potential model of Scalesia pedunculata carbon sequestration through restoration efforts in agricultural fields of Galapagos
Source: PLoS One. 2024 May 16;19(5):e0302680. doi: 10.1371/journal.pone.0302680 (PMC11098518; doi:10.1371/journal.pone.0302680)
Supplement: Attachment — Submitted filename: Reply to reviewers.docx [file pone.0302680.s006.docx]

**Journal Requirements:**

When submitting your revision, we need you to address these additional requirements.

1. Please ensure that your manuscript meets PLOS ONE's style requirements, including those for file naming. The PLOS ONE style templates can be found at 
<https://journals.plos.org/plosone/s/file?id=wjVg/PLOSOne_formatting_sample_main_body.pdf> and 
<https://journals.plos.org/plosone/s/file?id=ba62/PLOSOne_formatting_sample_title_authors_affiliations.pdf>

The style was amended

2. Thank you for stating the following financial disclosure: "This research was funded by the “COmOn Foundation (Code: 1-63D-663)"
Please state what role the funders took in the study.  If the funders had no role, please state: "The funders had no role in study design, data collection and analysis, decision to publish, or preparation of the manuscript." 
If this statement is not correct you must amend it as needed. 
Please include this amended Role of Funder statement in your cover letter; we will change the online submission form on your behalf.

Indeed, the funders had no role in study design, data collection and analysis, decision to publish, or preparation of the manuscript.

3. Please provide a complete Data Availability Statement in the submission form, ensuring you include all necessary access information or a reason for why you are unable to make your data freely accessible. If your research concerns only data provided within your submission, please write "All data are in the manuscript and/or supporting information files" as your Data Availability Statement.

All data was uploaded to a public GitHub.

https://github.com/GalapagosVerde2050/Plos-One---Carbon

4. We note that Figure 2 in your submission contain [map/satellite] images which may be copyrighted. All PLOS content is published under the Creative Commons Attribution License (CC BY 4.0), which means that the manuscript, images, and Supporting Information files will be freely available online, and any third party is permitted to access, download, copy, distribute, and use these materials in any way, even commercially, with proper attribution. For these reasons, we cannot publish previously copyrighted maps or satellite images created using proprietary data, such as Google software (Google Maps, Street View, and Earth). For more information, see our copyright guidelines:

<http://journals.plos.org/plosone/s/licenses-and-copyright>.

The copyright holder of the image is the Charles Darwin Foundation 🡪 <https://geodata.fcdarwin.org.ec/catalogue/#/dataset/249>

Updated reply: I have asked the IT department about the license that we have in the CDF, and they updated the metadata on the file. The data comes originally from NASA – Aster data, which has CC Zero

At the CDF these digital elevation models are reprocessed to fit the Galapagos Island profile.

I hope this explain better the copyright of the image.

 
We require you to either (1) present written permission from the copyright holder to publish these figures specifically under the CC BY 4.0 license, or (2) remove the figures from your submission:

a. You may seek permission from the original copyright holder of Figure 2 to publish the content specifically under the CC BY 4.0 license.  
 
We recommend that you contact the original copyright holder with the Content Permission Form (<http://journals.plos.org/plosone/s/file?id=7c09/content-permission-form.pdf>) and the following text:
“I request permission for the open-access journal PLOS ONE to publish XXX under the Creative Commons Attribution License (CCAL) CC BY 4.0 (<http://creativecommons.org/licenses/by/4.0/>). Please be aware that this license allows unrestricted use and distribution, even commercially, by third parties. Please reply and provide explicit written permission to publish XXX under a CC BY license and complete the attached form.”

Please upload the completed Content Permission Form or other proof of granted permissions as an ""Other"" file with your submission.

In the figure caption of the copyrighted figure, please include the following text: “Reprinted from [ref] under a CC BY license, with permission from [name of publisher], original copyright [original copyright year].”

b. If you are unable to obtain permission from the original copyright holder to publish these figures under the CC BY 4.0 license or if the copyright holder’s requirements are incompatible with the CC BY 4.0 license, please either i) remove the figure or ii) supply a replacement figure that complies with the CC BY 4.0 license. Please check copyright information on all replacement figures and update the figure caption with source information. If applicable, please specify in the figure caption text when a figure is similar but not identical to the original image and is therefore for illustrative purposes only.
The following resources for replacing copyrighted map figures may be helpful:

USGS National Map Viewer (public domain): <http://viewer.nationalmap.gov/viewer/>
The Gateway to Astronaut Photography of Earth (public domain): <http://eol.jsc.nasa.gov/sseop/clickmap/>
Maps at the CIA (public domain): <https://www.cia.gov/library/publications/the-world-factbook/index.html> and <https://www.cia.gov/library/publications/cia-maps-publications/index.html>
NASA Earth Observatory (public domain): <http://earthobservatory.nasa.gov/>
Landsat: <http://landsat.visibleearth.nasa.gov/>
USGS EROS (Earth Resources Observatory and Science (EROS) Center) (public domain): [http://eros.usgs.gov/#](http://eros.usgs.gov/)
Natural Earth (public domain): <http://www.naturalearthdata.com/>

5. Please include captions for your Supporting Information files at the end of your manuscript, and update any in-text citations to match accordingly. Please see our Supporting Information guidelines for more information: <http://journals.plos.org/plosone/s/supporting-information>.

[Note: HTML markup is below. Please do not edit.]

Reviewers' comments:

Reviewer's Responses to Questions

**Comments to the Author**

1. Is the manuscript technically sound, and do the data support the conclusions?

The manuscript must describe a technically sound piece of scientific research with data that supports the conclusions. Experiments must have been conducted rigorously, with appropriate controls, replication, and sample sizes. The conclusions must be drawn appropriately based on the data presented.

Reviewer #1: Partly

Reviewer #2: No

2. Has the statistical analysis been performed appropriately and rigorously?

Reviewer #1: Yes

Reviewer #2: Yes

3. Have the authors made all data underlying the findings in their manuscript fully available?

The [PLOS Data policy](http://www.plosone.org/static/policies.action#sharing) requires authors to make all data underlying the findings described in their manuscript fully available without restriction, with rare exception (please refer to the Data Availability Statement in the manuscript PDF file). The data should be provided as part of the manuscript or its supporting information, or deposited to a public repository. For example, in addition to summary statistics, the data points behind means, medians and variance measures should be available. If there are restrictions on publicly sharing data—e.g. participant privacy or use of data from a third party—those must be specified.

Reviewer #1: No

Reviewer #2: Yes

4. Is the manuscript presented in an intelligible fashion and written in standard English?

PLOS ONE does not copyedit accepted manuscripts, so the language in submitted articles must be clear, correct, and unambiguous. Any typographical or grammatical errors should be corrected at revision, so please note any specific errors here.

Reviewer #1: No

Reviewer #2: Yes

5. Review Comments to the Author

Please use the space provided to explain your answers to the questions above. You may also include additional comments for the author, including concerns about dual publication, research ethics, or publication ethics. (Please upload your review as an attachment if it exceeds 20,000 characters)

Reviewer #1: The background of the work is explained nicely with specific objectives and a hypothesis but the specific research questions are not mentioned. I have some queries in the methodology section please refer to the manuscript wherein I have given my comments in the sticky notes embedded in the manuscript itself. Please conclude your study adding a separate heading i.e., conclusion. Please see my comment in the manuscript. Results are nicely explained with tables and illustrations but the discussion have a scope of improvement in a way that can be compared with earlier works based on its advantages and disadvantages including the correctness or goodness of fit of your models as compared to earlier studies. Some sentences were too long and thus not fully comprehended. Some I have highlighted. Please use shorter and simple sentences for easy understanding of the readers. Overall the study was interesting and has novelty and will help in with informed decision making for carbon management with native species in the Galapagos island.

We checked the provided pdf and considered all reviewer suggestions deeply.

Reviewer #2: I have the following queries about the manuscript:

1. At line number 101 the author had mentioned the area to be Humid Tropics. However, the rainfall mentioned is 400 - 500 mm which does not seem to be a Humid range but is attuned toward semi-arid tropics.

Indeed, the information was not accurate. We updated it with a new reference. Line 129.

2. Line Number 180 -180: Survival probabilities were used. Why the author had not used the exact survival, that would have verified the impact of technology used.

We included a short explanation for this decision. Lines 256-257.

3. Line 231- 235: I believe it should have been the soul aim to find why the variation in survival happened or growth variation was varied due to adoption of the planting techniques.

Albeit these questions are proper, the experiment did not focus on cause-effect relationships. On the contrary, on the discussion we have speculate about potential way in which technologies work (lines 338-340; 361-362). The overall results show that most technologies did not have bigger differences in survival for the period analysed, that’s why a suggestion (now on the discussion: line xxx) is to increase the amount of restoration technologies and evaluated period.

4. Overall the manuscript lacked consistency in setting the target and observing the data as per the treatments set.

In the new version we included research questions, rewrite some sentences and improve the streamlining according to the other reviewer comments, so we hope the new version is more consistent.

6. PLOS authors have the option to publish the peer review history of their article ([what does this mean?](https://journals.plos.org/plosone/s/editorial-and-peer-review-process#loc-peer-review-history)). If published, this will include your full peer review and any attached files.

If you choose “no”, your identity will remain anonymous but your review may still be made public.

**Do you want your identity to be public for this peer review?** For information about this choice, including consent withdrawal, please see our [Privacy Policy](https://www.plos.org/privacy-policy).

Reviewer #1: **Yes:** Professor (Dr) Sumit Chakravarty

Reviewer #2: No
